# One Health Investigation into Mpox and Pets, United States

**DOI:** 10.3201/eid3010.240632

**Published:** 2024-10

**Authors:** Clint N. Morgan, Natalie M. Wendling, Nicolle Baird, Chantal Kling, Leah Lopez, Terese Navarra, Gracie Fischer, Nhien Wynn, Leslie Ayuk-Takor, Brandy Darby, Julia Murphy, Rachel Wofford, Emma Roth, Stacy Holzbauer, Jayne Griffith, Ali Ruprecht, Charlalynn Harris, Nadia Gallardo-Romero, Jeffrey B. Doty

**Affiliations:** Centers for Disease Control and Prevention, Atlanta, Georgia, USA (C.N. Morgan, N.M. Wendling, N. Baird, C. Kling, L. Lopez, T. Navarra, G. Fischer, N. Wynn, S. Holzbauer, C. Harris, N. Gallardo-Romero, J.B. Doty);; Oak Ridge Institute for Science and Education, Oak Ridge, Tennessee, USA (L. Lopez, T. Navarra, G. Fischer);; DC Department of Health, Washington, DC, USA (L. Ayuk-Takor);; Virginia Department of Health, Richmond, Virginia, USA (B. Darby, J. Murphy);; Council of State and Territorial Epidemiologists, Applied Epidemiology Fellowship Program, Atlanta (R. Wofford);; Tennessee Department of Health, Nashville, Tennessee, USA (R. Wofford, E. Roth);; Minnesota Department of Health, Saint Paul, Minnesota, USA (S. Holzbauer, J. Griffith, A. Ruprecht);; Chenega Enterprise Systems and Solutions, Chesapeake, Virginia, USA (C. Harris)

**Keywords:** Mpox, monkeypox virus, viruses, zoonoses, companion animals, pets, dogs, cats, One Health, orthopoxvirus, DNA contamination, household, United States

## Abstract

Monkeypox virus (MPXV) is zoonotic and capable of infecting many mammal species. However, whether common companion animals are susceptible to MPXV infection is unclear. During July 2022–March 2023, we collected animal and environmental swab samples within homes of confirmed human mpox case-patients and tested for MPXV and human DNA by PCR. We also used ELISA for orthopoxvirus antibody detection. Overall, 12% (22/191) of animal and 25% (14/56) of environmental swab samples from 4 households, including samples from 4 dogs and 1 cat, were positive for MPXV DNA, but we did not detect viable MPXV or orthopoxvirus antibodies. Among MPXV PCR-positive swab samples, 82% from animals and 93% from environment amplified human DNA with a statistically significant correlation in observed cycle threshold values. Our findings demonstrate likely DNA contamination from the human mpox cases. Despite the high likelihood for exposure, we found no indications that companion animals were infected with MPXV.

Before 2022, the primary mode for monkeypox virus (MPXV) transmission was known to be zoonotic, and only limited human-to-human transmission was documented (*1*,*2*). Human MPXV infections resulting in mpox disease were hypothesized to be the result of direct or potentially indirect contact with infected wild mammals in Central and Western Africa (*3*,*4*). Our understanding of the potential for human-to-human spread of MPXV considerably broadened in the spring of 2022 (*5*,*6*). During that time, variant of clade II MPXV (clade IIb) was found in to be transmitted via direct contact among human populations and spreading primarily through sexual networks outside of mpox endemic regions (*5*,*6*).

Given the zoonotic origin and reported broad host-range of MPXV, efforts to understand and limit potential human-to-animal transmission are ongoing (*4*,*7*). The Centers for Disease Control and Prevention (CDC) provides guidance that persons with mpox stop or avoid contact with animals and that animals should be kept away from potentially infectious lesion material, objects, or surfaces (*8*). Mpox patients are generally urged by public health agencies to isolate at home unless hospitalization is clinically necessary (*9*,*10*). A person with mpox is considered infectious throughout their illness and until lesions have fully healed with new skin underneath; therefore, public health officials recommend that mpox patients isolating at home take proper infection control measures to prevent spread of infectious particles throughout the home (*11*–*13*). Unless infected persons take measures to completely isolate or reduce transmission potential, companion animals in close contact with mpox patients and their environments could be at higher risk for MPXV exposure than other mammal species, warranting special concern and investigation.

As of July 2024, no cases of MPXV infection or mpox disease had been confirmed in common domestic animals, such as dogs and cats, during the current global outbreak or any past outbreaks. One study in July 2022 described a 4-year-old dog in France that had been living and co-sleeping with 2 mpox case-patients (*14*). In that study, MPXV DNA was identified in swab samples from the dog’s skin and surface of mucosal lesions and in anal and oral swab samples (*14*). However, follow-up investigations suggested that the animal was not infected with MPXV (*15*). A similar case was documented in Brazil in August 2022, when a 5-month-old dog had lesions that were MPXV-positive by quantitative PCR (*16*). Whether viral DNA detection was a result of MPXV infection in those animals or the result of environmental contamination due to close contact with infected humans is unclear. We conducted a One Health investigation in the United States to assess the susceptibility of companion animals to mpox and the risk for reverse-zoonotic transmission within households.

## Methods and Materials

### Study Population

The CDC Multi-National Mpox Response’s One Health Team worked in collaboration with state and local jurisdictions to investigate the susceptibility of companion animals to MPXV infection. As part of that effort, CDC and state public health investigators collected blood samples from companion animals and swab specimens from companion animals and animal-associated objects. CDC tested swab and serum specimens via real-time PCR, orthopoxvirus (OPXV) serology, and viral culture. All animals tested were companion animals in a residence of a person with probable or confirmed mpox while the person was infectious. Animal sampling occurred within 21 days of any direct contact with the ill person before the person recovered (Table 1). 

**Table 1:** Summary of variables coded from household and questionnaire data used in a One Health investigation into mpox and pets, United States*

HH no.	Animal ID	DIS	CSI	AOA†	Household area, ft^2^‡	CCR	Rodents in household	Wildlife around household	SXDS	No. days exposure§	
										Before sampling	After recovery
1	DC-001	2	N	None	500–825	N	Y	Y	N	22	1
	DC-002	2	N	None					N	22	1
2	DC-003	6	N	None	500–825	N	N	N	N	22	13
3	DC-004	0	N	None	1,500–2,000	Y	N	N	Y	11	0
4	DC-005	5	Y	Walks	900–1,350	N	N	Y	Y	9	0
5	DC-006	6	N	Walks	500–825	Y	N	Y	N	16	6
6	DC-007	0	N	Walks	500–825	Y	N	Y	Y	28	0
7	DC-008	6	Y	Yard	1,500–2,000	Y	N	Y	Y	17	0
	DC-009	6	Y	Yard					Y	17	0
8	VA-001	2	N	Yard	1,500–2,000	Y	Y	Y	N	14	10
	VA-002	3	N	Yard					N	14	10
9	VA-003	2	N	Walks	500–825	Y	N	N	N	20	2
10	VA-004	7	Y	None	500–825	N	N	N	Y	18	0
11	VA-005	2	N	None	500–825	Y	N	N	N	12	12
	VA-006	2	N	None					N	10	12
12	MN-001	4	Y	None	500–825	Y	N	Y	Y	7	0
13	MN-002	3	Y	None	1,500–2,000	Y	Y	Y	Y	13	0
14	MN-003	5	Y	Walks	500–825	Y	N	Y	Y	46	0
15	MN-004	3	Y	Yard	1,500–2,000	Y	N	Y	Y	12	0
16	MN-005	6	N	None	1,500–2,000	N	Y	Y	N	26	17
	MN-006	6	N	None					N	26	17
	MN-007	6	N	None					N	26	17
	MN-008	6	N	None					N	26	17
	MN-009	6	N	Yard					N	26	17
	MN-010	6	N	Yard					N	26	17
17	TN-001	2	N	None	900–1,350	Y	N	Y	N	36	12
	TN-002	2	N	None					N	36	12
18	TN-003	2	N	None	1,500–2,000	Y	N	Y	N	25	14
	TN-004	2	N	None					N	25	14
19	TN-005	6	N	Yard	2,000–3,500	N	Y	Y	N	36	7
	TN-006	6	N	Yard					N	36	7
	TN-007	3	N	Yard					N	36	7
20	TN-008	5	N	Walks	1,500–2,000	Y	N	Y	N	UNK	27
21	TN-009	3	Y	None	900–1,350	N	N	Y	Y	17	0

*Variables relate to companion animals and peridomestic wildlife and include duration of potential monkeypox virus exposure to the animal within the household of the mpox case in relation to the date of sampling. AOA, animal outdoor activity; CCR, contact change with animals reported after mpox diagnosis in household member; CSI, co-sleeping with animal while owner infectious; DIS, direct interaction score comprised of the sum of all reported interaction types involving direct contact; HH, household; ID, identification; SXDS, mpox case-patient with symptoms during sampling; UNK, unknown. †Considered as none (pet not allowed outside), walks (periodic or frequent supervised walks outside), or yard (periodic or prolonged unsupervised outdoor activity). ‡Based on data reported or estimated, the approximate area range is displayed for increased anonymity. §Cumulative days potentially exposed to mpox.

During July 2022–March 2023, we conducted sample collection in the District of Columbia, Virginia, Minnesota, and Tennessee, USA. After the initial sampling timepoint, we attempted follow-up sampling from all households 3–4 months later to collect animal serum samples and assess postexposure or postinfection immune responses.

### Questionnaire and Consent

State and local public health personnel from the District of Columbia, Virginia, Minnesota, and Tennessee assisted with the study by interviewing mpox cases in their jurisdictions and requesting their voluntary participation in the study. After a person gave verbal consent to participate, they were provided with a survey questionnaire and consent forms. The questionnaire ascertained details and a timeline of the human case, the animal’s health condition, general household information, types of contact between the person with mpox and the animal or animals in the household, and information about wild or domestic animals in and around the household. This project was reviewed by CDC clearance, cleared for human subjects, and determined to be nonresearch public health surveillance that did not require submission to the CDC institutional review board (project no. 0900f3eb81f79d72).

### Swab Sample Collection

We performed all animal handling and sampling procedures in accordance with the approved CDC Institutional Animal Care Use Committee protocol (no. DOTMULX3183), in collaboration with state public health agencies, and with written consent of the animal’s owner. We collected a standardized set of polyester swab (Puritan, https://www.puritanmedproducts.com) samples from the animal’s dorsum fur, ventral abdomen, oral cavity, and anorectal area under supervision of the owner. We sampled animal lesions, if present. We also collected animal-associated environmental (AAE) specimens from objects and surfaces often used by the animal.

### Sample Processing and PCR

We processed swab samples by using the swab extraction tube system (SETS; Roche, https://www.roche.com) with 400 μL of phosphate-buffered saline; after DNA extraction, we tested all samples for MPXV DNA by real-time PCR using an MPXV clade II–specific assay (*17*). In addition, we tested samples for human DNA by using the RNase-P PCR assay, which is used as an endogenous control when testing human specimens (*18*). We calculated Pearson correlation coefficients to assess the relationship between cycle threshold (Ct) values of MPXV clade II PCR–positive (Ct values <37) and RNase-P–reactive (Ct values <40) samples.

### Viral Culture

We tested all PCR-positive swab samples for viable virus via cell culture by adding an aliquot of swab eluate to BSC-40 cell monolayers in T-25 flasks. We used an inoculation volume of 50 μL +25 μL, depending on available eluate volume. We incubated flasks at 35.5°C in an atmosphere of 6% CO_2_ in Roswell Park Memorial Institute medium (*19*). We incubated and observed flasks <14 days or until ≈100% of monolayer showed cytopathic effect, following methods and media supplements described previously (*11*). To control the overgrowth of bacteria or fungi in T-25 flasks, we added penicillin/streptomycin, amphotericin B, and gentamicin to the cell culture medium. If we detected any bacterial or fungal contamination, we performed 4 cycles of medium replacement to wash the monolayers and repeated this process as needed to prevent overgrowth.

### Blood Collection and Serologic Testing

We attempted blood collection from all cooperative animals for which the owner provided consent. We collected <3 mL of blood from 20/34 animals during initial sampling and 21/25 animals during follow-up sampling. We cleaned the external venipuncture site with 90% ethanol and used a syringe or vacutainer needle for blood collection. For dogs and 1 rabbit, we collected blood via the cephalic or lateral saphenous veins. For cats, we collected blood via the jugular or medial saphenous veins. We stored and transported blood tubes at 4°C–20°C before centrifugation, after which we transferred serum into 2-mL cryotubes and stored at temperatures of at least –20°C until laboratory testing. We conducted a modified ELISA on all serum samples to determine presence of OPXV IgG antibodies, as previously described (*20*,*21*). We tested serum samples at a dilution of 1:100 by using microtiter plates coated with purified vaccinia virus (Dryvax strain) and using the A/G protein as the secondary antibody at a 1:10,000 concentration and developed plates for 25 minutes.

### Data Analysis

 When referring to animal swab samples, we defined prevalence as the proportion of total swabs collected from each animal from which we detected either MPXV DNA or RNase-P (RNP) by PCR. When referring to AAE samples, we defined prevalence as the proportion of total swabs collected from the AAE samples within that animal’s household that were MPXV-positive or RNP-positive. We also referred to detection of RNase-P via PCR as presence of human DNA.

For each animal, we calculated the duration of exposure, defined as cumulative number of days before sampling that an infectious owner had direct contact with the animal, including durations where direct contact was not reported but the animal was still sharing a common space with a person with mpox. Duration of exposure represented the total period that infectious lesion material (crusts or exudates) or other infectious particles were potentially shed or transferred within the home, to which the animal potentially had contact, either directly or via fomites.

We investigated factors reported in questionnaires that could affect animal MPXV exposure (Table 1). Those factors included whether the owner was symptomatic during time of sampling (coded SXDS); the degree of animal outdoor activity (coded AOA), which we stratified by none (no outdoor activity), walks (periodic or frequent supervised walks outside), and yard (allowed in yard or outside unsupervised frequently or for prolonged periods); co-sleeping with the animal while the owner was infectious (coded CSI); and a score comprised of the sum of all reported interaction types between animals and humans that involved direct contact (coded DIS), which included cuddling, hugging, petting, kissing, co-sleeping, sharing food, and grooming (Table 1).

We compared bivariate correlation coefficients among variables compiled from questionnaire data or diagnostic testing. We used SPSS Statistics 27 (IBM, https://www.ibm.com) to compute Pearson correlation coefficients. We performed 2-tailed tests of significance and considered p values of <0.05 or <0.01 statistically significant, as applicable.

## Results

Overall, we sampled 34 individual companion animals from 21 households: 24 domestic dogs, 9 domestic cats, and 1 domestic rabbit (Table 2). The age of the animals ranged from 4 months to 16 years; 22 were male and 12 were female. All but 1 household had a single human mpox case; the other household had 2 cases. We collected a total of 191 swab specimens from animals and 56 AAE specimens. If excess blood was available, we opportunistically tested select blood specimens via PCR, including 10 whole blood specimens preserved in EDTA and 1 blood clot. At examination, we observed skin lesions in 6 dogs and 1 cat, and lesion features and locations varied.

**Table 2:** Animal and environment sampling and diagnostic testing data from a One Health investigation into mpox and pets, United States*

Household no.	Animal ID	Species	Sex	Lesions sampled	Prevalence of animal sample PCR positivity†				Prevalence of environment sample PCR positivity		Serum timepoints
					MPXV	RNP	Ct		MPXV	RNP	
1	DC-001	Dog	M	N	0.0	0.8	ND		0.0	0.8	NC
	DC-002	Dog	M	N	0.0	0.6	ND		0.0	0.8	NC
2	DC-003	Cat	M	N	0.0	0.2	ND		0.0	0.3	1
3	DC-004	Dog	F	N	0.0	0.0	ND		0.0	0.5	NC
4	DC-005	Dog	M	Y	0.0	0.4	ND		0.0	0.7	1
5	DC-006	Dog	F	Y	0.0	0.3	ND		0.0	0.5	1
6	DC-007	Dog	M	N	0.0	0.4	ND		0.0	1.0	1
7	DC-008	Dog	F	N	0.0	0.6	ND		0.0	0.0	2
	DC-009	Dog	M	N	0.0	0.2	ND		0.0	0.0	2
8	VA-001	Dog	M	N	0.0	0.0	ND		0.0	0.0	1
	VA-002	Dog	M	Y	0.0	0.0	ND		0.0	0.0	2
9	VA-003	Dog	M	Y	0.7	0.6	35.4		1.0	0.7	1
10	VA-004	Cat	F	Y	0.2	0.3	36.4		1.0	1.0	NC
11	VA-005	Dog	M	Y	0.5	0.5	33		1.0	1.0	1
	VA-006	Dog	M	N	0.7	0.5	34.5		1.0	1.0	3
12	MN-001	Dog	F	N	0.5	0.6	34.4		1.0	1.0	NC
13	MN-002	Rabbit	M	N	0.0	0.2	ND		0.3	0.7	2
14	MN-003	Dog	M	Y	0.0	0.3	ND		0.0	0.3	2
15	MN-004	Dog	F	N	0.0	0.3	ND		0.0	1.0	2
16	MN-005	Cat	M	N	0.0	0.2	ND		NC	NC	2
	MN-006	Cat	M	N	0.0	0.4	ND		NC	NC	NC
	MN-007	Cat	M	N	0.0	0.0	ND		NC	NC	NC
	MN-008	Cat	M	N	0.0	0.0	ND		NC	NC	1
	MN-009	Dog	F	N	0.0	0.2	ND		NC	NC	2
	MN-010	Dog	M	N	0.0	0.5	ND		NC	NC	2
17	TN-001	Cat	M	N	0.0	0.2	ND		0.0	1.0	1
	TN-002	Cat	F	N	0.0	0.2	ND		0.0	1.0	2
18	TN-003	Dog	M	N	0.0	0.4	ND		0.0	0.7	1
	TN-004	Cat	F	N	0.0	0.4	ND		0.0	1.0	NC
19	TN-005	Dog	F	N	0.0	0.0	ND		0.0	0.0	2
	TN-006	Dog	F	N	0.0	0.3	ND		0.0	0.0	2
	TN-007	Dog	M	N	0.0	0.0	ND		0.0	0.0	2
20	TN-008	Dog	M	N	0.0	0.2	ND		0.3	0.7	1
21	TN-009	Dog	F	N	0.0	0.0	ND		0.0	0.0	2

*Ct, cycle threshold value; MPXV, monkeypox virus DNA; NC, no specimens collected; ND, not done; RNP, RNase-P DNA. †Proportion of total swab specimens that were MPXV or RNP positive.

### PCR for Animal Samples

Samples collected from 5 individual animals (4 dogs, 1 cat) from 4 households were MPXV-positive; 2 of the dogs shared a household. Total animal swab MPXV positivity was 12% (22/191); 21 MPXV-positive swabs were from dogs, and 1 was from a cat (Table 3). All MPXV-positive animals also had >1 sample with an RNP-positive test result. Ct values of MPXV-positive samples were 25.2–36.7 (mean 34.5). Results of specific sample types collected were 29% (4/14) for skin lesions, 16% (6/37) for ventral skin or fur, 12% (4/33) for dorsal fur, 11% (4/35) for periocular area, 8% (3/36) for anorectal area, and 3% (1/36) for oral.

**Table 3:** PCR results for monkeypox virus clade II and RNase-P DNA assays from swab samples of companion animals and animal-associated objects and surfaces during a One Health investigation into mpox and pets, United States

Swab samples	Monkeypox virus, no. (%)													RNase-P, no. (%)	
	Dogs			Cats			Rabbit			All					
Total	Positive	Total	Positive	Total	Positive	Total	Positive	Negative	Positive	Negative
Animal	140	21 (15)		47	1 (2)		4	0		191	22 (12)	169 (88)		18 (82)	42 (25)
Objects and surfaces	42	10 (24)		11	3(27)		3	1 (33)		56	14 (25)	42 (75)		13 (93)	24 (57)
Totals	182	31 (17)		58	4 (7)		7	1 (14)		247	36 (15)	211 (85)		31 (86)	66 (31)

Among animal MPXV-positive specimens, 82% were RNP-positive, whereas 25% of the MPXV DNA–negative specimens were RNP-positive (Table 3). Ct values of MPXV-positive specimens that were RNP-positive positively correlated (p<0.01). In animal specimens, 18% (4/22) were MPXV-positive and RNP-negative, and positive Ct values (range 35.3–36.1) were near the upper limit of detection (Ct 37) for the assay. We did not detect MPXV DNA in any of the blood specimens tested via MPXV PCR. In addition, MPXV DNA prevalence in animal samples alone and when combined with AAE specimens significantly correlated with RNP prevalence in those same samples (p<0.05).

### AAE PCR

We collected AAE specimens from 20/21 households, predominately from animal beds or bedding, toys, and food and water dishes. Among households, 29% (6/21) were positive for MPXV DNA, as were 25% (14/56) of collected specimens, 93% (13/14) of which were positive for MPXV and RNP (Table 3). In those same samples, AAE MPXV DNA prevalence positively correlated with human DNA prevalence (p<0.05). Of the 4 households with MPXV-positive animal swab specimens, all had MPXV-positive AAE swabs with Ct values of 29.9–35.9 (mean 32.8). For AAE specimens that were MPXV- and RNP-positive, the MPXV and RNase-P Ct values were significantly correlated (p<0.01). Of all AAE specimens, 66% (37/56) were RNP-positive, of which 82% (9/11) of specimens with Ct values <37 were in the 4 households with MPXV-positive AAE and animal swab samples.

### Viral Culture and Serology

We attempted viral culture from all specimens with Ct values <36 (n = 31), and all were negative with no signs of cytopathic effect. Three specimens from 2 dogs had bacterial contamination causing destruction of monolayer by day 6 or 7 postinfection, despite mitigating steps or retesting, and the harvested culture media tested negative by MPXV-specific PCR. In addition, all initial (n = 20) and follow-up (n = 22) serum specimens collected were ELISA-negative, and we detected no OPXV IgG. For 1 dog that had samples with the lowest MPXV Ct values, we collected 2 follow-up samples 2 months apart. Of the 5 animals that had MPXV-positive swab specimens, 3 did not have blood sampled at the initial timepoint due to noncompliance or aggression, and 3 were not available at the postexposure sampling timepoint.

### Questionnaire Analysis

In total, 32% (11/34) of animals had preexisting health issues and 5 animals had preexisting skin lesions. In addition to the 5 animals with skin lesions that developed before owner symptom onset (all sampled), 2 additional animals had lesions that developed after owner symptom onset. We observed and sampled those lesions during the initial sampling visit, and 1 animal had skin and fur, periocular, and anorectal specimens that were PCR-positive for MPXV DNA, but we did not detect MPXV DNA from the lesion specimen, and serology results also were negative.

In total, 33% (7/21) of households reported no contact change with their animals. Reported types of changes in animal interactions included reducing frequency of interactions (9/21), stopping interactions (8/21), use of PPE during interactions (6/21), and relocating or isolating the animal (4/21); 1 household reported relegating animal care to uninfected persons outside the household. However, all but 1 household reported >1 type of direct contact activity with each animal after the MPXV-positive human in the household had symptoms develop (Table 1). 

Households comprised apartments (n = 11) or single-family homes (n = 10), and approximate size range was 500–3,500 ft^2^ (Table 1). We observed a significant negative correlation between household size and prevalence of either MPXV (p<0.05) or human DNA (p<0.01) in animal samples and human DNA prevalence in environmental samples (p<0.01). Apart from human DNA prevalence, household size, and environmental MPXV prevalence, we observed no other statistically significant relationships for other variables potentially influencing prevalence of MPXV DNA in animal samples.

## Discussion

CDC advises that persons with mpox should avoid contact with animals, including pets, until lesions have fully healed to prevent potential virus spillback. That recommendation is because of uncertainty regarding susceptibility of companion animals to MPXV (*9*). If MPXV-infected persons cannot avoid contact with pets within the household, practicing appropriate infection control will prevent further exposure potential. In most households we visited, recommended quarantine and infection control procedures were not consistently followed.

Despite MPXV-positive swab specimens detected on the skin or fur of dogs and cats and in associated environmental samples, no dogs or cats with live virus or antibodies detected have been reported globally. In 2 cases outside of the United States in which MPXV DNA was detected in dogs (*14*,*16*), apart from apparent skin lesions, no other signs of infection were reported in the animals, including virus cultured from samples or OPXV antibodies detected by serology after additional investigation (*15*).

In our household study, skin lesions in 7 animals were the only observable clinical features that were potentially consistent with mpox disease. However, 5 animals exhibited lesions before owner symptom onset, and the 2 animals with skin lesions that were observed after owner symptom onset were negative for MPXV by PCR. Only 1 animal had MPXV-positive lesions sampled, a dog with lesion swab samples collected from a grouping of 3 large lesions on its rear leg, and the average Ct value of samples was 25.2. After further testing to consider potential DNA contamination from the owner, that sample also had the lowest average RNase-P Ct value (29.3) of all samples tested. In addition, that dog’s lesions were reported to have formed before symptom onset in the owner, culture attempts from that and all other samples were negative, and OPXV antibodies were not detected during any timepoint tested. Therefore, after reviewing all the data, we did not consider this animal a confirmed mpox case.

All animals with MPXV-positive samples in this study also had RNP-positive specimens collected, indicating the presence of human DNA. The statistically significant correlation of MPXV- and RNP-positive samples, MPXV PCR results showing high Ct values indicating low viral DNA loads, and the lack of viable virus or antibodies in the collected samples strongly suggest that observed lesions or scabs in these animals were not the result of MPXV infection. In addition, from our knowledge of MPXV pathology, an MPXV lesion would most likely produce high viral loads and at levels higher than for other sample types (*22*).

As reported in other household environmental sampling studies, MPXV DNA can be widely detected in indoor or household settings (*11*,*12*,*23*–*25*). In this study, we found that households with smaller shared spaces were significantly correlated with both MPXV and human DNA prevalence, suggesting that the risk for MPXV exposure could be higher in smaller living quarters. Given the capability of MPXV DNA to disseminate within the household of a person with mpox, and after consideration of the PCR results detailed here, persons with mpox, not the companion animals, likely were the source of the MPXV DNA we detected in the household.

The potential for contamination from either direct contact with a person with mpox or indirect exposure to materials containing MPXV DNA should be considered when interpreting results of PCR testing from companion animals. In addition, case definitions should consider potential extraneous contamination and require more than a PCR-positive result from an animal to be considered a confirmed animal mpox case (*26*). Contamination should also be considered as a reason for a positive PCR result and false-positive results in humans with nonspecific lesions who have potentially had contact with an mpox case-patient.

MPXV infection in companion animals, if they are suitable hosts, is uncharacterized; clinical signs, viral shedding, and duration of infectious period are unknown. Thus, although unlikely, given the limits of our sampling design, it is possible that an infected animal escaped detection in our study. However, the overall PCR and serologic evidence best fits the hypothesis that the MPXV DNA detected in animal samples submitted for PCR testing is a result of DNA contamination from the infected human within the household. 

More work is needed to determine the susceptibility of companion animals to clade IIb MPXV. Thus, CDC still recommends that companion animal owners with mpox limit their interactions with their pets while infectious, particularly if they are sharing smaller living spaces. That precautionary measure is recommended until more information is available about the susceptibility of common mammalian companion animal species to mpox. 

In conclusion, no strong evidence yet exists to suggest that common companion animals, such as dogs or cats, are susceptible to infection with clade IIb MPXV. Given high likelihood for exposure among most of these animals, the paucity of evidence indicating infection might indicate resistance to infection. Nonetheless, to prevent further viral spread and potential evolution and establishment of new endemic areas, during public health emergencies caused by emerging zoonotic diseases, responders should apply a One Health approach to investigate potential spillback of human infections to animals, including pets. 
